# Electrospinning Nanoparticles-Based Materials Interfaces for Sensor Applications

**DOI:** 10.3390/s19183977

**Published:** 2019-09-14

**Authors:** Shan Zhang, Zhenxin Jia, Tianjiao Liu, Gang Wei, Zhiqiang Su

**Affiliations:** 1 State Key Laboratory of Chemical Resource Engineering, Beijing Key Laboratory of Advanced Functional Polymer Composites, Beijing University of Chemical Technology, Beijing 100029, China; 2017200436@mail.buct.edu.cn (S.Z.); 2017200441@mail.buct.edu.cn (Z.J.); 2016200362@mail.buct.edu.cn (T.L.); 2 College of Chemistry and Chemical Engineering, Qingdao University, Qingdao 266071, China; 3 Faculty of Production Engineering, University of Bremen, D-28359 Bremen, Germany

**Keywords:** electrospinning, nanoparticles, hybrid materials, interfaces, sensors

## Abstract

Electrospinning is a facile technique to fabricate nanofibrous materials with adjustable structure, property, and functions. Electrospun materials have exhibited wide applications in the fields of materials science, biomedicine, tissue engineering, energy storage, environmental science, sensing, and others. In this review, we present recent advance in the fabrication of nanoparticles (NPs)-based materials interfaces through electrospinning technique and their applications for high-performance sensors. To achieve this aim, first the strategies for fabricating various materials interfaces through electrospinning NPs, such as metallic, oxide, alloy/metal oxide, and carbon NPs, are demonstrated and discussed, and then the sensor applications of the fabricated NPs-based materials interfaces in electrochemical, electric, fluorescent, colorimetric, surface-enhanced Raman scattering, photoelectric, and chemoresistance-based sensing and detection are presented and discussed in detail. We believe that this study will be helpful for readers to understand the fabrication of functional materials interfaces by electrospinning, and at the same time will promote the design and fabrication of electrospun nano/micro-devices for wider applications in bioanalysis and label-free sensors.

## 1. Introduction

Nanoparticles (NPs) exhibit good catalytic, conductive and optical properties, and have showed broad applications in the fields of materials science, biomedicine, tissue engineering, energy storage, environmental science, and sensor [1,2,3,4,5]. Recently, more and more attention have been focused on the fabrication of functional Nanomater. by using various NPs for assembling high-performance sensors and biosensors [6,7,8,9]. However, NPs usually have high specific surface energy and tend to agglomerate, leading to weaken or disappeared performance [10,11]. To solve this problem, various strategies such as surface modification of NPs [12,13], self-assembly [14,15,16], template-based synthesis [17,18], electrospinning [19,20], and others have been utilized to create materials interfaces with desired structure and function, in which the electrospinning technique has attracted increasing attention due to its high efficiency, simple operation, and low cost. Compared with other fiber formation processes, electrospinning provides a simpler and more economical production process, and the nanofibers obtained by electrospinning are thinner in size with higher surface area and obvious void structure [21,22].

The fabricated materials interfaces of NPs with polymers can effectively avoid the agglomeration of NPs, and greatly improve the ability of matrix materials to participate in electron transfer and transport, extending their applications in sensors and biosensors. In addition, the wrapping of NPs in a matrix can protect the stability of NPs and facilitate their recycling. Both factors make NPs the ideal building blocks to fabricate electrospun sensor devices. In recent years, the applications of electrospinning technique to introduce NPs into/onto polymer fibers to prepare composite fibrous materials interfaces for sensors have been reported widely [23,24]. 

In this work, we would like to present recent advance in the fabrication and sensor applications of the NP-based materials interfaces through electrospinning. In the second part, the strategies for fabricating materials interfaces based on electrospinning various NPs, such as metal NPs (MNPs), oxide NPs, alloy/metal oxide NPs, and carbon NPs, are demonstrated and discussed firstly; in the third part, we introduced the fabrication of various sensor devices by using the NP-based materials interfaces, and presented their applications as sensors for electrochemical, electric, fluorescent, colorimetric, surface-enhanced Raman scattering (SERS), photoelectric, and chemo-resistance sensors. It is expected this review will be helpful for readers to understand the design and fabrication of functional materials interfaces for various applications beyond sensors and biosensors.

## 2. Electrospinning NP-Based Material Interfaces

In this section, the electrospun materials interfaces based on various NPs, such as MNPs, oxide NPs, alloy/metal oxide NPs, and carbon NPs, are introduced.

### 2.1. MNP-Based Interfaces

The preparation of MNPs/polymer composite fibers by electrospinning can be divided into blending, post-modification, and post-treatment methods. The blending method refers to the process that MNPs are mixed with the polymer solution to form a uniform precursor solution, and then the mixed solution is directly electrospun to form various materials interfaces. This method has the advantages of simple preparation and high yield, and has been widely used to prepare fluorescent and electrochemical sensing interfaces. In post-modification, nanofibers are prepared by electrospinning, and then metal nanoparticles are adsorbed or modified onto the nanofibers to obtain the MNP-based interface. The post-treatment method is based on the blending method to conduct post-treatment on the obtained MNP-based interfaces, such as calcining, so as to obtain the interface of the new structure.

**Figure 1:**
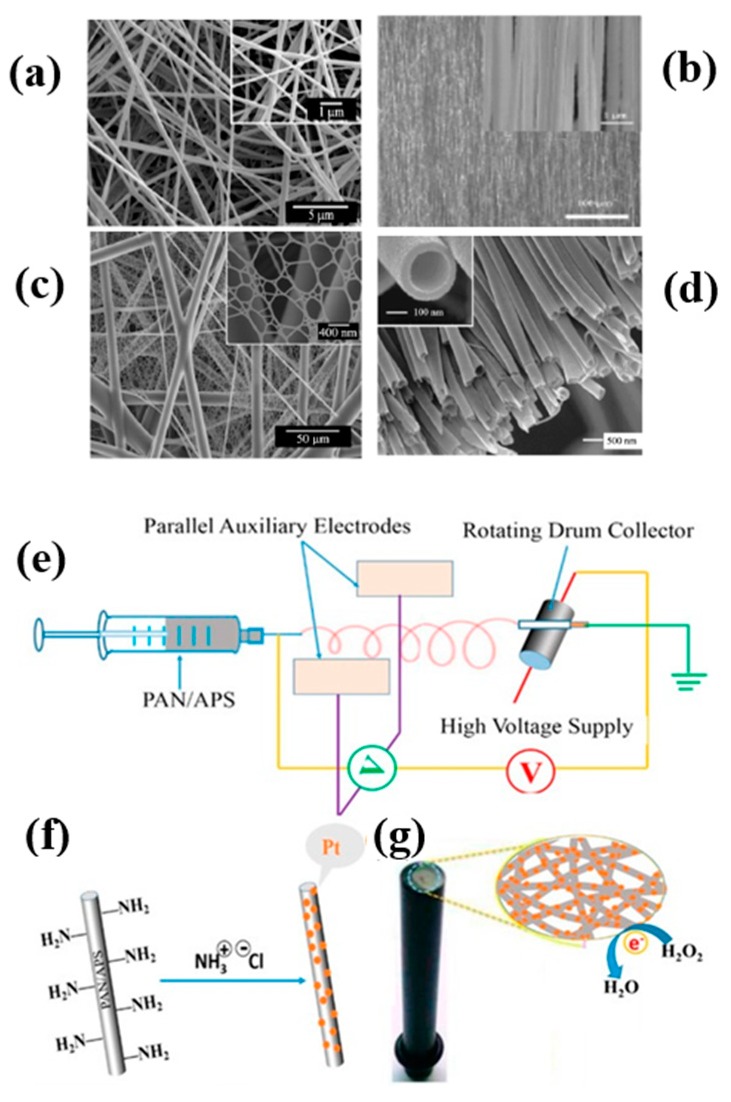
(**a**) SEM image of irregular fibers, [26] copyright 2010 Elsevier; (**b**) SEM image of highly oriented fibers, [27] copyright 2007 Scitation; (**c**) SEM image of nano-nets, [28] copyright 2011 Royal Society of Chemistry; (**d**) SEM image of hollow fiber, [29] copyright 2004 American Chemical Society; (**e**) model of a home-made electrospinning instrument for the synthesis of polyacrylonitrile/3-aminopropyltriethoxysilane (PAN/APS) hybrid nanofibers; (**f**) electrostatic assembly mechanism of platinum NPs (PtNPs) along amino-modified PAN nanofibers; (**g**) PAN–PtNPs hybrid nanofibrous membrane-modified electrode and the potential sensing mechanism of hydrogen peroxide (H_2_O_2_) [33], copyright 2017 MDPI.

Yang et al. reported the in situ fabrication of Ag nanoparticles (AgNPs)/polyacrylonitrile (PAN) hybrid fibers by electrospinning [25]. It was found that the structure and properties of AgNPs were stable under high voltage field, and the addition of AgNPs increased the diameter and conductivity of composite fibers to some extent, making the insulated polypropylene (PPP) nanofibers to form the semiconductor. The structure of the fiber determines the properties of the fiber, so the development of functional fiber must begin with the regulation of the structure of the fiber, which mainly includes the regulation of the structure of the fiber aggregate and single fiber. As shown in Figure 1a, the fiber aggregation can be divided into irregular fibers and highly oriented fibers according to their fiber orientation. In addition, nano-nets is a novel fiber structure with good stability and mechanical properties. According to its specific morphology and structure, a single fiber can be divided into solid, hollow, and core-shell structures with different properties [26,27,28,29]. Compared with the traditional intercalation and template-synthesis methods, electrospinning can produce nanofibers with more diverse and fine structures. For example, by changing the nozzle structure, it is possible to obtain solid, hollow, core-shell structure of ultrafine fibers or spider network structure of two-dimensional (2D) fiber membrane. In addition, the single fiber, fiber bundle, highly oriented fiber, and irregular oriented fiber membrane can be obtained by designing different receiving devices. Therefore, the prepared composite nanofibers have improved properties. For example, Khan et al. used different collection devices to prepare five polyvinylpyrrolidone/gold NPs (PVP/AuNPs) composite nanofibers with different diameters by electrospinning [30]. The good stability and dispersion of AuNPs in nanofibers were proved by scanning electron microscopy (SEM) and transmission electron microscopy (TEM). Due to the high biodegradation and biocompatibility of polyvinylpyrrolidone (PVP) and the antibacterial function of AuNPs [31,32] the fabricated nanofibers were expected to be implanted into the body’s axons, providing nerve signals from the damaged site to anywhere else in the nerve axon. It was found that the PVP/AuNPs with diameter of 0.2 mm, whose capacity of potential voltage up to 89 mV, are the most suitable tubes for the axon application. However, in practical production, using different nozzles and receivers also means higher cost and technical difficulty, which also limits its application in industrial fields.

The post-modification method developed from the blending method is also a common method to prepare polymer spinning and modify some functional substances on the spinning surface to obtain functional materials. Compared with the blending method, the post-modification method is suitable for more kinds of polymer solutions and can change the functional molecules modified on the spinning as needed. Recently, we reported a combination of PAN and platinum NPs (PtNPs) into composite nanofibers for high performance electrochemical sensors [33]. As shown in Figure 1, PAN was electrospun with 3-aminopropyltriethoxysilane (APS) together as precursor materials, which were then further modified by the amino group to bind with PtNPs through the electrostatic interaction. 

In addition to polymer nanofibers, the combination of MNPs and carbon nanofibers (CNFs) also attracts great interest [20,34]. Compared with other materials, the super-small size of nanoparticles not only gives them unique properties, but also makes them in an energy unstable state due to their huge specific surface area and surface energy [35]. The distance between nanoparticles is very short, so the van der Waals force, hydrogen bond, chemical bond and other interactions between nanoparticles are strong, which leads to the tendency of agglomeration of nanoparticles due to mutual adsorption [36]. Due to the self-polymerization tendency of MNPs, various carriers have been used to maintain their stability in order to retain their excellent catalytic activity. However, the commonly used 2D and three-dimensional (3D) materials are not conducive to electron transfer and diffusion, thus affecting the activity of catalysts supported by them. Compared with 2D or 3D materials, one-dimensional (1D) nanofibers have extremely high orientation and length-diameter ratio, which enables rapid and stable one-way electron transport [37]. CNFs have the advantages of low density, high specific modulus, high specific strength, and high electrical conductivity. Their large surface area is also conducive to the dispersion of MNPs, which makes them have profound application potential in the field of catalysis and sensing. Unlike polymer nanofibers, CNFs can only be prepared by post-treatment. Post-treatment refers to the preparation of electrostatic spinning with a mixed solution as the precursor and the preparation of spinning after high temperature calcination. Compared with other methods, the fibers prepared by the post-treatment method have smaller diameter and rougher surface. For example, Zhang et al. reported a new palladium NPs (PdNPs)/CNFs that can be used to detect hydrazine [38]. Using the dimethylformamide (DMF) solution of PAN and Pd(acetate)_2_ (Pd(Ac)_2_) as precursors, PdNPs/CNFs have been fabricated successfully by electrospinning. The disadvantages of this method are also obvious. The post-treatment process is complicated, and the calcining does not meet the requirements of environmental protection.

### 2.2. Oxide NP Interfaces

Oxide NPs, which can be divided into non-metallic oxide NPs (non-MONPs) and metal oxide NPs (MONPs), can also be combined with nanofibers to obtain composite fibers with corresponding functions [39,40,41]. Compared with MNPs, oxide NPs are easier to prepare and have lower cost. In addition, they also have excellent optical, electrical, and magnetic properties, as well as good biocompatibility, which attracts a wide interest of researchers [42,43,44].

**Figure 2:**
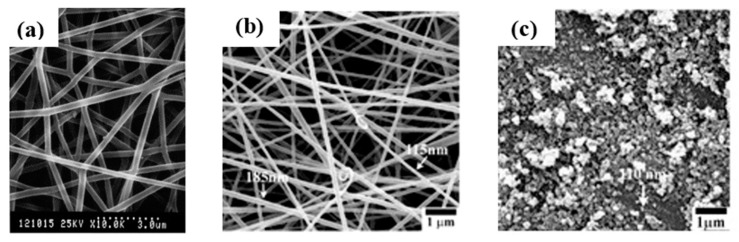
Fabrication of oxide nanoparticles (NPs) by electrospinning: (**a**) Scanning electron microscopy photographs of various polyvinyl alcohol (PVA)/silica fibers with silica content of 22wt% [48], copyright 2003 Elsevier Ltd; (**b**) scanning electron microscopy images of zinc acetate (Zn(OAc)_2_)/cellulose acetate (CA) composite nanofibers containing 10wt% Zn(OAc)_2_; (**c**) NPs from calcination of (a) in air at 500 °C for 5 h [49], copyright 2008 American Chemical Society.

Silicon dioxide (SiO_2_) is the most commonly used non-MONPs [45]. Composite fibers prepared by electrospinning technology have the advantages of high specific surface area, high porosity, and good biocompatibility. Early studies have been focused on the use of sol-gel technology to combine with electrospinning to prepare oxide/polymer composite nanofibers by blending inorganic sols and organic polymers [46,47]. This method has been widely used in the preparation of composite nanofibers due to its simple preparation process and low cost. For instance, Shao et al. prepared SiO_2_/PVA (polyvinyl alcohol) composite fiber materials by blending the SiO_2_ gel and PVA solution [48]. First, they obtained silica gel by the hydrolyzed polycondensation of phosphoric acid and TEOS (tetraethoxysilane). After 5 h, they added 10 wt% PVA solution and reacted for 12 h to obtain the PVA/silica composite gel. The solution was the electrospun to obtain the corresponding silica/PVA composite nanofibers, whose structure is shown in Figure 2a. The obtained results indicated that the thermal stability of SiO_2_/PVA composite fiber materials is significantly improved than that of PVA fiber materials. However, the reaction time required by the sol-gel method is long and the prepared nanofibers are not uniform.

Compared with the earlier sol-gel method, the blending method has aroused the interest of the majority of researchers because of its high uniformity of products and controllable preparation process. Oxide NPs are dispersed directly into the polymer solution and then electrospun to create non-metallic oxide/polymer composite nanofibers. In a typical study, Kanehata et al. dispersed SiO_2_ NPs with diameters of 15 nm, 50 nm, and 100 nm into the PVA solution for electrospinning, and prepared SiO_2_/PVA composite fiber materials [50]. It was found that the addition of SiO_2_ NPs made the surface of the composite become rough and present grain-like process. With the increase of particle diameter, the diameter of composite fiber decreases correspondingly due to the change of the solution viscosity.

Compared with non-MONPs, MONPs have better electrical conductivity, and therefore they have shown wider applications in the fields of photocatalysis and biosensors [51,52,53]. For example, zinc oxide (ZnO) NPs have large specific surface area, high isoelectric point, excellent compatibility with proteins, biological cells, and other biological materials. It is safe and non-toxic, so it is suitable for the adsorption of proteins with low electrical point. Fang et al. prepared hollow ZnO nanospheres by the hydrothermal method [54]. As a semiconductor material, ZnO has a band gap of about 3.37 eV at room temperature. Under ultraviolet light with a wavelength of less than 378 nm, photogenerated hole-electron pairs can be generated, while positively charged holes have a strong ability to get electronic. It is a strong oxidant that can directly capture and oxidize the electrons in the organic matter, so as to achieve the effect of catalytic degradation of organic pollution [55]. Generally, the mixed solution of hydrolyzable metal salt and polymer is used as the precursor for spinning, and the corresponding nanofibers can be obtained by the post-treatment method, which is mainly treated by calcining. For example, Liu et al. prepared composite nanofibers by electrospinning with zinc acetate (ZnAc)/cellulose acetate (CA) as the precursor, which were converted into ZnO nanofibers and NPs after calcining. In their experiment, ZnAc was added to a 20 wt% CA in DMF/acetone solution, which was then electrospun to obtain composite nanofiber membranes. Zn(OH)_2_/cellulose nanofibers are then obtained by hydrolysis of the membrane in an alkali solution, which was calcining at 500 °C for 5 h to transform into ZnO nanofibers. The structure of it is shown in Figure 2b,c [49]. This material has a high photocatalytic activity, which can catalyze the photodegradation of dye molecules rhodamine B and acid fuchsia under the irradiation of visible light.

In addition to ZnO NPs, other metal oxides such as indium oxide(In_2_O_3_) [56], nickel oxide(NiO) [57], and aluminum oxide (Al_2_O_3_) [58] can also be used to prepare composite nanofibers by electrospinning. In addition, the work on oxide composite fiber is not limited to the preparation and structural characterization of new materials. Recently, more studies have been focused on the functional development of materials. The post-modification method has also been applied in the preparation of metal oxide/polymer composite nanofibers. For example, Lu et al. prepared titanium oxide(TiO_2_)/polypyrrole (PPy) coaxial fibers by in situ polymerization on the surface of TiO_2_ fibers prepared by electrospinning [59]. The coaxial fiber has both the conductive property of PPy and the surface photoelectric property of TiO_2_, so it is expected to be applied in the fields of photoelectric transformation. This fabrication strategy of this kind of TiO_2_/PPy composite fiber provides a new idea for the development of similar materials. That is to say, a new functional material is obtained by preparing two known functional materials into a new NP interface through electrostatic spinning, so as to retain the original function and have a new structure. To this end, researchers need to further study the relationship between the interface structure and properties of nanoparticles.

In addition to coaxial fibers, other structures of electrospinning nanofibers have also been studied. Since the structure of fibers determines the properties of fibers, the development of functional fibers must start from regulating the structure of fibers [60,61,62]. 

### 2.3. Alloy/metal Oxide NP Interfaces

It is well known that different NP materials have different characteristics [63]. With the development of electrochemical biosensors, single Nanomater. are often difficult to meet their performance requirements [64,65]. In particular, in the field of sensing application, the single noble metal nanometer has such problems as serious agglomeration, high production cost, low utilization rate of single nanometer material and serious oxidation deactivation [66]. Therefore, using two or more Nanomater. to design and prepare composite nanofibers with high activity and stability by utilizing the interface effect and synergistic effect between NPs has become another new development trend [67,68,69,70,71]. In the construction of nanoscale sensing interfaces, the metal alloy sensing interface has shown amazing performance in reducing intrinsic and charge transfer resistance, protecting highly active particles and improving the synergistic effect of materials, which has been widely concerned by scholars in various fields [72,73,74,75,76].

**Figure 3:**
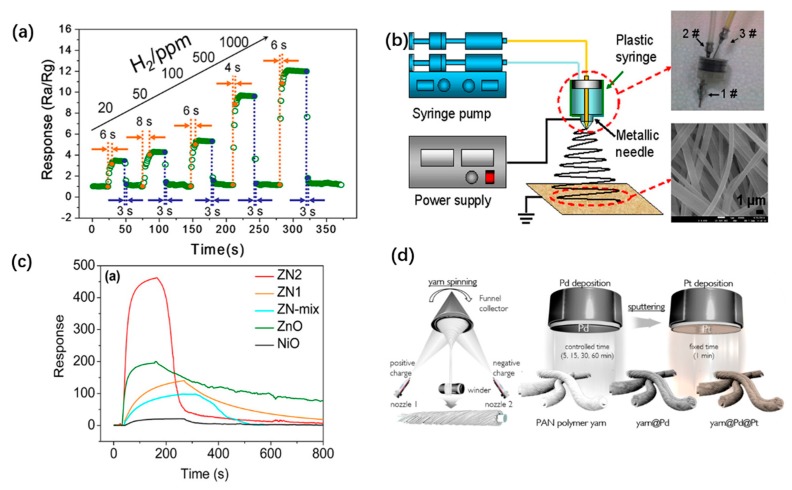
Fabrication of alloy/metal oxide (MO) interfaces: (**a**) Dynamic H_2_ sensing transients of all sensors with different loading levels at % to 20, 50, 100, 500, and 1000 ppm in turn [77], copyright 2017 MDPI; (**b**) schematic diagram of the experimental blending method setup. The insets are the actual photo of the coaxial dual spinnerets (the top one) and the SEM image of the precursor nanofibers (the below one); (**c**) response transients of different gas sensors to 20 ppm H_2_S at 215 °C [78], copyright 2012 American Chemical Society; (**d**) the synthesis of the yarn/Pd and yarn/Pd/Pt via yarn spinning followed by a sputter-deposition process, copyright [79] 2019 American Chemical Society.

Wang et al. proposed a simple preparation method for the Pd^0^-SnO_2_ composite nano sensor interface by the blending method [77]. Pd^2+^-loaded SnO_2_ composite fibers were prepared by pre-prepared SnCl_2_ and PdCl_2_ blends through the electrospinning. After that, the composite fibers were heat-treated to obtain Pd^2+^-loaded SnO_2_ composite fibers, which were finally prepared under the reduction of hydrazine hydrate. In Pd^0^-SnO_2_ composite nanofibers, the height of the depletion barrier increases due to the transfer of electrons from the N-type material to the P-type material. When the material is exposed to the detected phase, electrons are trapped, and the P-type material feeds back electrons to the N-type material. Through the interaction of the interface, the resistance of the device changes, and the detection sensitivity is greatly improved. At the optimal Pd content, the lower detection limit of Pd^0^-SnO_2_ composite nanofibers reached a very low level (20 ppb), achieving faster response (4−13 s) and shorter recovery (3−9 s) at room temperature as shown in Figure 3a. However, in a mixed gas environment, the selectivity of composite nanofiber sensor needs to be further tested.

Xu et al. used a co-fibril post-treatment method to construct a nickel oxide/zinc oxide (NiO/ZnO) composite nano–sensing interface with a coaxial heterostructure [78]. The preparation process mainly includes the preparation of precursor of electrospinning, blending method electrospinning mixture and heat treatment, as shown in Figure 3b. By the blending method, the outer shell structure containing ZnO NPs can be continuously attached to the core material surface containing NiO NPs to form a heterogeneous coaxial structure. The core-shell structure is sensitive to H_2_S in the gas phase and has fast response. It is worth noting that the core-shell structure has fast recovery time (~124 s) compared with the single ZnO gas sensor, which is only 1/10 of the recovery time of the single ZnO gas sensor, as shown in Figure 3c, and has excellent stability and repeatability. In addition, by using the post-modification method, the sensor particles with high activity can be loaded on the surface of the fiber material through in situ synthesis, sputtering, and other methods, which can effectively construct the sensor interface and enhance the sensing performance of the material. 

In another study, Kim et al. prepared Pt polymer nanofiber yarn coupled with nanograined Pd/Pt by using electrospinning yarn polymer nanocomposites and subsequent direct current (DC) magnetron sputtering method, as shown in Figure 3d [79]. This method can stably attach PdNPs to the fiber surface, and effectively construct the shell and core structure of Pt-Pb to form a sensing interface that can quickly recover itself. There is no doubt that the sensor interface of hybrid nanofibers with alloy NPs will gradually replace the traditional hybrid sensor of single precious metal.

### 2.4. Carbon NP Interfaces

Carbon materials are an important part of the field of nanometer materials, known as the 21st century, one of the most important nanomaterial, because in optics, electromagnetism, and superior mechanical and thermal properties, in chemistry, materials, biology, medicine, and many other areas show the inviting application prospect, caused a great response to science, gradually become a hot topic of scientific research workers [80]. As an important branch of nanosensors, the construction of carbon NPs sensing interface has attracted the attention of researchers from various countries due to its good electrical conductivity, wide potential window, stable emission property, large specific surface area, rich surface functional groups, high temperature resistance, and chemical corrosion resistance [81]. Different types and structures of carbon NPs sensing interfaces are constructed by combining different electrostatic spinning processes, which further widens the research scope of nano-particle sensing interfaces and enables the acquisition of sensors with faster response, high stability, and reusable applications [82].

**Figure 4:**
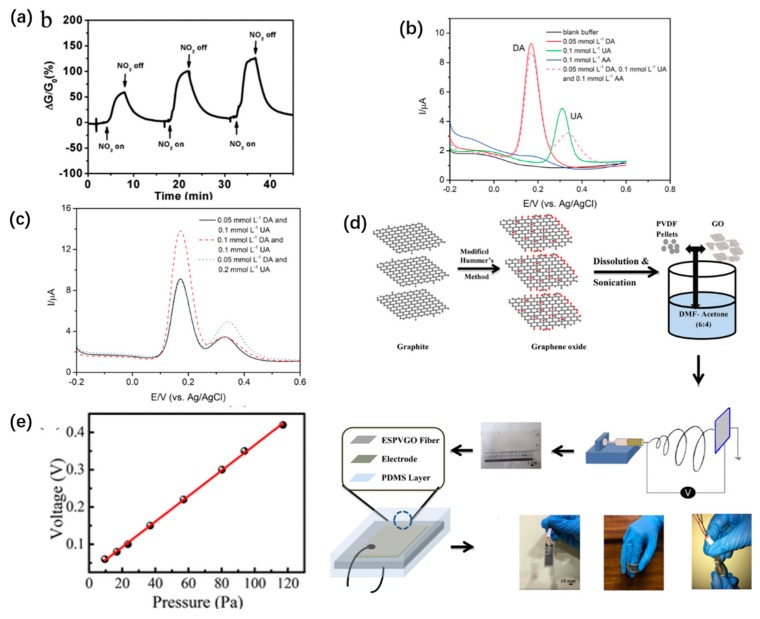
Fabrication of carbon NP interfaces: (**a**) Plot of response versus time for an reduced graphene oxide/polymer composite nanofibers (rGO/P NFs)-based sensor upon exposure to NO_2_ gas with a concentration of 500 ppb, 1 ppm, or 2 ppm [84], copyright 2014 American Chemical Society; differential pulse voltammetry (DPV) curves for the polyamide 6/poly allylamine hydrochloride- multiwalled carbon nanotubes (PA6/PAH_MWCNTs) electrode: (**b**) For detection of Dopamine (DA), ascorbic acid (AA), and uric acid (UA), separately, and then in mixtures with the concentrations indicated in the inset; (**c**) for simultaneous detection of DA and UA in concentrations ranging from 0.05 to 0.2 mmol L^−1^ [90], copyright 2015 American Chemical Society. (**d**) schematic route for the fabrication of graphene-based piezo- and pyro-electric nanogenerator (GPPNG); (**e**) open-circuit output voltage of the GPPNG under different applied pressure [91], copyright 2019 American Chemical Society.

Using the post-modification method to self-assemble the active NPs on the fiber surface to construct the sensor interface of carbon NPs, the active particles can be exposed to the fiber surface directly and fully, and the sensitivity of the sensor components can be improved [83]. Yuan et al. impregnated the prepared polymer nanofibers and their substrates in the dispersion solution of graphene oxide (GO) prepared by the modified Hummer method [84]. Since the nanofibers were positively charged in the aqueous phase, negatively charged GO could self-assemble on the surface of the fibers to form go composite nanofibers. After that, GO nanofibers were reduced to graphene nanofibers. Under the NO_2_ atmosphere with a concentration of 500 ppb, the response time of reduced GO (rGO) composite nanofibers was less than 3 min and the recovery response time was less than 6 min, as shown in Figure 4a. However, the authors did not explain the enhancement mechanism properly. In addition, Guan et al. used this method to adsorb carbon nanotubes on the surface of PA66 nanofibers to prepare carbon nanotube/PA66 composite nanofiber materials. CNT with high electrical conductivity endows the composite fiber with electrical conductivity, and PA66 with high flexibility improves the toughness of the composite.

By comparing previous reports of graphene NPs compared sensors, such as graphene [85], GO [86], graphene nano network [87], AuNPs hybrid rGO [88], rGO/metal [89], and rGO/metal oxide [17], it can be found that sensor sensitivity of the fabricated sensors can be improved 2–30 times after chemical modification of rGO [87]. It is worth noting that the test results show that the sensor interface prepared by this method reaches 150 ppb. After that, Mercante et al. also used the method after modification to prepare the electrochemical dopamine sensor with high sensitivity by loading multi-wall graphene nanotubes on the surface of polymer nanofibers [90]. The prepared limit of the sensor reached 0.15 µM with high stability and repeatability. In addition, the sensor prepared by this method has very high sensor selectivity. The sensor selectivity is shown to be able to accurately react to detected substances in complex environments. DA in the biological environment main molecular potential interference of ascorbic acid (AA) and uric acid (UA), even under the condition of the above human serum concentration, AA detection signal is so low that is not enough to cause the attention of people, under the environment of DA and UA, can still clearly separate the peak of the DA, as shown in Figure 4b,c, enabling high precise selection that reflects the tested material.

The blending method is also one of the important methods to construct the sensing interface of carbon NPs. The blending method can construct carbon NPs with sensing activity in the fiber cavity, increasing the mechanical stability of the active particles. Roy et al. first prepared GO using the Hummer modified method. Subsequently, the prepared go was dispersed in PVDF/DMF/acetone solution to prepare the blend. PVDF/GO nanofibers are then prepared by dispersing the blends using an electrostatic spinning device, as shown in Figure 4d [91]. The prepared rGO/PVDF composite nanometer sensor has a high-pressure sensitivity of 4.3 v/kPa and the detection limit is as low as 10 pa. On the other hand, rGO/PVDF composite nanofibers have better thermoelectric properties, with the highest output power density up to ~1.2 nw/m^2^, as shown in Figure 4e

### 2.5. Summary

As shown in Table 1, NPs, which can be divided into MNPs, oxide nanoparticles, alloy NPs, and carbon NPs, can be combined with nanofibers through electrostatic spinning. By electrospinning, NPs can be combined with nanofibers to obtain different and controllable interfaces with special structures. The extremely large surface of nanofibers provides sufficient attachment sites for NPs, and as one-dimensional Nanomater., they have extremely high orientation and length-diameter ratio, which is also conducive to the rapid electron transport. This interface not only retains the excellent optical, electrical, magnetic properties, and good biocompatibility of NPs, but also effectively avoids the agglomeration of NPs induced by their own surface free energy, greatly improving the stability of the interface. In addition, the diversity and controllability of the nanofiber assembly prepared by electrostatic spinning also make the functional expression of this interface have greater application potential, which is mainly manifested in the field of sensing. 

## 3. Sensor Applications of Electrospun NP-based Material Interfaces

In this section, we would like to demonstrate recent advances in the fabrication of electrospun NP-based materials interfaces for electrochemical, electronic, fluorescent, colorimetric, SERS, and other kind of sensor applications. 

### 3.1. Electrochemical Sensors

Electrochemical sensor refers to a device that can qualitatively or quantitatively detect substances by measuring the electrical and electrochemical properties of target molecules or substances to be measured, and then convert the perceived signals into identifiable electrical signals proportional to the concentration of target substances through specific transducers [92,93,94]. According to the different output signals can be divided into current sensors, conductance sensors, and potential sensors. The current sensors are used to analyze the current through external circuit as the signal of the sensor when the chemical reaction occurs, so as to realize the purpose of detecting chemical substances; the conductance sensors take the change of the conductance of the electrolyte solution after the oxidation or reduction of the measured substance as the output signal of the sensor to analyze, so as to realize the material detection; the potential sensors determine the concentration of a substance by measuring the balance potential of the electrode. 

In general, the selectivity of the sensor, which means the sensor can effectively identify the measured substance in the presence of complex test environment or multiple interfering substances, is one of the bases for the establishment of the sensor. Only by improving the selectivity of the sensor can the stability and reliability of the sensor test results be guaranteed. Thus, the relevant physical and chemical information of the measured substance can be obtained accurately. The specificity of the sensor means that the sensor only outputs signals for the measured substance, so the test results are specific and accurate. Electrochemical sensors have been developed as one of the most active fields in the analytical field and widely used in various fields of work and life. Compared with traditional electrode modified materials, the advantages of electrostatic spinning nanofibers, such as small structure size and large specific surface area, play a good role in promoting the performance of improving the sensitivity, response speed and selectivity of sensors. Therefore, they gradually show great potential for the application in the field of sensors. In recent years, electrostatic spinning fibers have been widely used in gas sensors, moisture sensors, especially electrochemical sensors.

**Figure 5:**
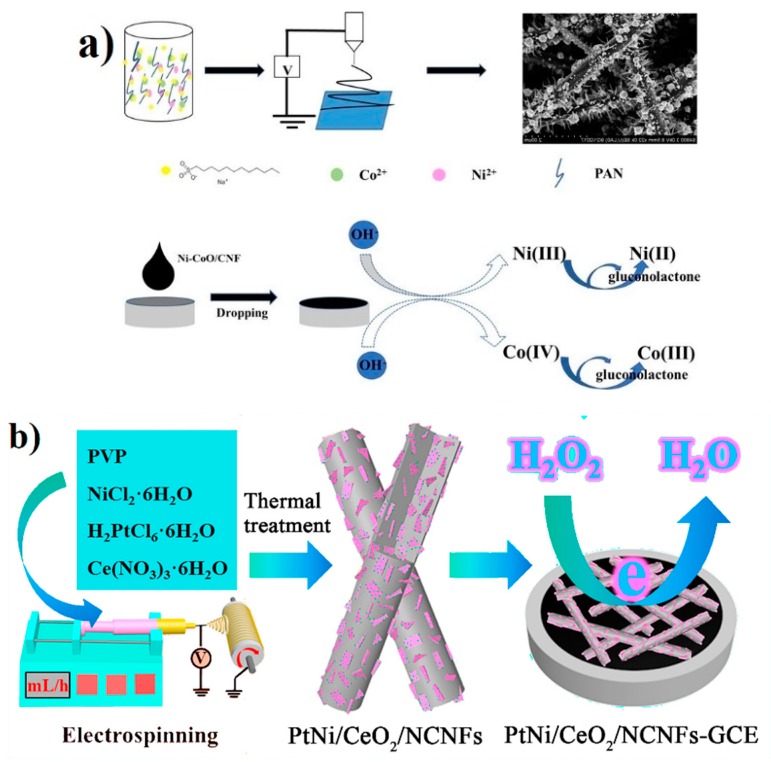
Electrospun NP interfaces for electrochemical sensors: (**a**) Experiment process and mechanism of carbon nanofibers (CNFs) loaded with nickel (Ni)/cobaltous oxide (CoO) NPs [95], copyright 2019 Elsevier Ltd; (**b**) preparation and sensing principle of platinum-nickel alloy(PtNi)/cerium oxide(CeO_2_)/n-doped carbon nanofibers(NCNFs) [63], copyright 2019 Elsevier Ltd.

There are two main methods for the application of nanofibers in sensors: The first one is electrospinning functional polymers such as polyacrylic acid (PAA) and polyaniline (PANI ) to obtain nanofibers with inductive function. Electricity spinning nanofibers are served as the sensing elements of sensors directly. Mei et al. prepared CNFs loaded with nickl(Ni)/cobaltous oxide (CoO) NPs by electrospinning, and used the anion surfactant equilibrium adsorption method to make the NPs grow uniformly in situ on the CNFs [95]. As shown in Figure 5a, by adjusting and comparing experimental conditions, such as applied potential and solution concentration, they obtained the composite with the best performance. The sensor can be used for the quantitative detection of glucose with a wide linear range (0.25–600 μM), low detection limit (0.03 μM), and good repeatability, and the electrochemical response after 30 days was retained at 86.8%. In addition, the amount of surfactant was found to have an impact on the surface morphology of the sensor, which in turn affected the sensitivity of glucose detection. Recently, Rezaei et al. developed a silver nanoparticle modified polyvinyl alcohol/chitosan (PVA/CS) composite nanofiber [96]. They first used the blending method to prepare PVA/CS nanofibers by electrospinning. Then, the silver ion is introduced by combining with the free amino group in chitosan, which forms nanoparticles on the fiber surface after reduction. During reduction, the presence of azopurine (AZA) affects the formation of silver nanoparticles, thereby reducing their plasma resonance intensity, which is linearly related to the concentration of AZA within a certain range. Therefore, this material can be used as a fast and sensitive AZA concentration probe with a wide linear range (0.14–2.88 μM) and a low detection limit (0.09 μM).

Apart from glucose [97,98,99,100], hydrogen peroxide (H_2_O_2_) plays an important role in the physiological and pathological processes of cell metabolism, growth, proliferation, development, and differentiation, aging and apoptosis, so its detection is also crucial [99]. As shown in Figure 5b, Guan et al. obtained platinum-nickel alloy/cerium oxide/n-doped carbon nanofibers (PtNi/CeO_2_/NCNFs) through electrospinning and calcination, in which PtNI/CEO_2_ was uniformly embedded in CNFs [63]. This special structure can initiate synergy and enhance its enzyme-like electrocatalytic activity, thus improving the detection sensitivity of H_2_O_2_. This sensor has a wide linear range (0.5 μM–15 nM), high sensitivity (345.0 μAmM^−1^cm^−2^), and low detection limit (0.025 μM), and can be used for the detection of H_2_O_2_ in cosmetics. In recent years, water pollution, especially heavy metal ion pollution, has attracted wide attention because of its great harm to human life and health. Therefore, sensitive and accurate heavy metal ion sensors have been widely studied. Chauhan et al. developed a ternary composite of silver nanoparticles-nylon 6 (PA6) electrospun nanofibers-cellulose nanowhiskers (CNW), which can be used as sensor parts for electronic tongue [101]. The conductivity of the sensor increases with the decrease of fiber diameter, and the sensor and frequency can be adjusted to detect different metal ions. The fabricated sensor exhibited a detection limit of 10 nM towards the selective detection of Pb^2+^ ions.

### 3.2. Electronic Sensors 

The toxic and harmful gases generated by decoration have seriously threatened people’s health and greatly affected the quality of life and work. In the face of all kinds of problems caused by poisonous and harmful gases, gas sensors emerge at the historic moment [102,103,104]. The typical gas sensors include electrochemical, thermal, and optical sensors, among which the electronic sensors have attracted more interest due to their high sensitivity, fast response, and good selectivity. 

The resistance sensor mainly uses semiconductor oxide or conductive polymer as the sensitive material. Through the change of resistance value of gas sensor with the change of gas content, it is possible to get the corresponding gas signal. In recent years, electrospinning 1D Nanomater. have made great progress in improving gas sensitivity due to their high specific surface area and fast mass transfer speed. Compared with the traditional structure, the semiconductor oxide with nanofiber structure can speed up the mass transfer of the detection material into and out of the reaction area, and the carrier is easier to transfer along the fiber axis, so that the resistance sensor has better detection performance.

**Figure 6:**
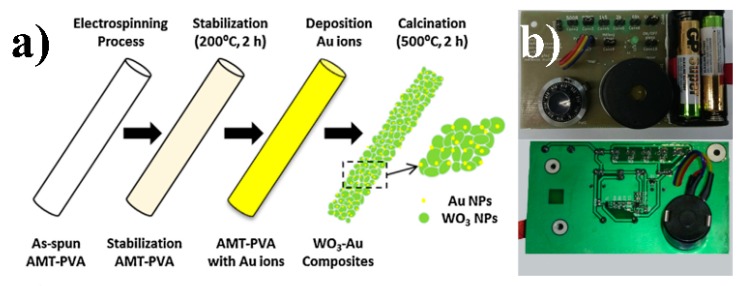
Fabrication of NP interfaces for electronic sensors: (**a**) The formation of tungsten oxide (WO_3_)-Au composites nanofibers [108], copyright 2015 Elsevier Ltd; (**b**) front and back side snapshots of proposed comparator [111], copyright 2016 Elsevier Ltd.

The single oxide semiconductor gas sensor is difficult to meet the practical requirements of good stability and mass production due to its weak nature [105]. The introduction of noble metal [106], transition metal [107], and other elements has also been proved to be another effective method to improve the new sensing ability. As shown in Figure 6a, Yang et al. prepared tungsten oxide (WO_3_) composite nanofibers modified with gold NPs by electrospinning and calcining with ammonium metamatellate hydrate (AMT) and PVA as precursors [108]. Among them, WO_3_ is a wide band gap (3.7 eV) metal semiconductor, which is often used as raw material for gas sensors, while precious metals such as palladium, platinum, and gold are considered as catalysts for gas activation [109]. In their experiments, composite nanofibers with different gold content were synthesized and their gas-sensitive properties were tested. By comparison, it was found that the response speed of WO_3_–Au-0.1 M sensor to n-butanol was 60 times that of pure WO_3_. In addition, the optimal operating temperature is lower than pure WO_3_, and the response time and recovery time are improved [110].

In addition to semiconductor oxides, nanofiber-structured conductive polymer materials not only have high specific surface area, chemical specificity, and adjustable conductivity, but also have good flexibility and processing properties, which are also ideal materials for resistance sensors. Currently, conductive polymers used in resistance sensors include PANI, PDA, PPy, and PT, among which polyaniline sensing materials are the most widely used. The reaction principle is based on the significant change of conductivity in the protonation and deprotonation process of PAN. The polyaniline nanotubes prepared by using polyvinyl alcohol electrospinning nanofibers as templates have the advantages of high specific surface area, small diameter, and high porosity, making the sensor display excellent sensing performance. Compared with PAN materials without template synthesis, PAN nanotube sensors show higher sensitivity and faster response to (C_2_H5)_3_N. In addition, the PAN sodium meter sensor also has good recovery. The structure of polyaniline sensor prepared by electrostatic spinning is shown in Figure 6b [111].

### 3.3. Fluorescent Sensors

Fiber optic sensors have attracted much attention recently due to their advantages such as probeless electrical contacts, no electromagnetic interference, and the multiplicity of individual network structures. Optical sensors based on electrospun nanofibers are among the research hotspots [33,112,113]. A fluorescent fiber is a fiber that absorbs energy upon irradiation of a specific wavelength of excitation light, enters an excited state, and immediately radiates energy outward in a fluorescent manner. Nano-fluorescent fibers prepared by electrospinning are mainly used in the fields of ion detection and adsorption, thin film sensors, and others. Fluorescence sensors are not only fast in response, high in sensitivity, but also highly selective. Commonly used fluorescent substances are classified into organic fluorescent substances and inorganic fluorescent substances [114].

**Figure 7:**
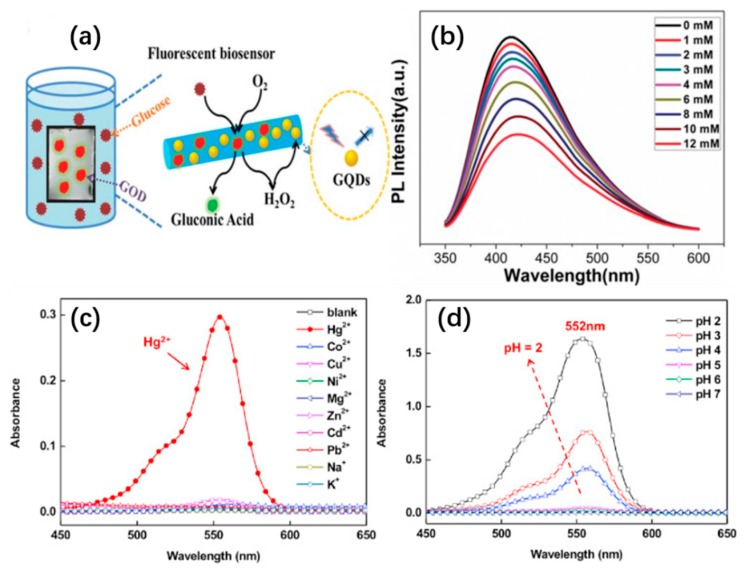
Fabrication of NP interfaces for fluorescent sensors: Fluorescent detection of glucose (**a**) the possible detection mechanism and (**b**) the fluorescence spectra of the PVA/graphene quantum (PVA/GQD) nanofibrous membrane in the presence of GQD and glucose of various concentrations [115], copyright 2014 American Chemical Society; (**c**) absorption spectra of (c) RhBN_2_AM (7 × 10^−3^ M in CH_3_OH) at various pH values and (**d**) RhBN_2_AM (2 × 10^−5^ M in CH_3_CN) in an aqueous solution (Hg^2^+,Co^2+^,Cu^2+^, Ni^2+^,Mg^2+^,Zn^2+^e^2+^,Pb^2+^,Na^+^, and K^+^;10^−4^ M Tris-HCl buffer at pH 7), respectively [117], copyright 2017 Elsevier Ltd.

Su et al. prepared a PVA/graphene quantum dot hybrid nanofiber membrane using graphene quantum dots, which was applied to the particle fluorescence sensor [115]. It was found that the fluorescence intensity of the nanofiber membrane changed linearly with the concentration of H_2_O_2_ due to the fluorescence quenching of graphene quantum dots caused by H_2_O_2_, as shown in Figure 7a,b. In addition, they loaded glucose oxidase onto graphene/PVA nanofiber membrane to achieve the detection of glucose with high sensitivity and selectivity [116]. In another case, Chen et al. reported the preparation of poly(HEMA-CO-NMA-RHBN_2_AM) by electrospinning, and the surface of the nanofiber was treated by chemical crosslinking to enhance the stability of the composite fiber in water. The resulting composite nanofibers have a variety of sensing functions and exhibit extremely high sensitivity in pH sensing and Hg^2+^ detection, as shown in Figure 7c,d. It is noteworthy that composite nanofibers have reversible switching properties and high selectivity. In non-acidic solution or aqueous solution without Hg^2+^, the fluorescence performance of the composite nanofiber sensor cannot be activated. However, in acidic solution or aqueous solution with Hg^2+^, the composite nanofiber sensor will gradually change color according to the concentration of the detected particles [117]. The nanocomposite fiber with fluorescence phenomenon can be observed by the naked eye, so that the ion detection can be visually observed. 

### 3.4. Colorimetric Sensors

The colorimetric chemical sensor is one of the photochemical sensors, and detects the concentration of the detected particles by visual observation and contrast by means of the sensing gene and the detected object by means of the change in hue [118]. Due to the high sensitivity of colorimetric chemical sensors, colorimetric chemical sensors have become one of the most important sensors for detecting substances in the order of ppm or ppb.

**Figure 8:**
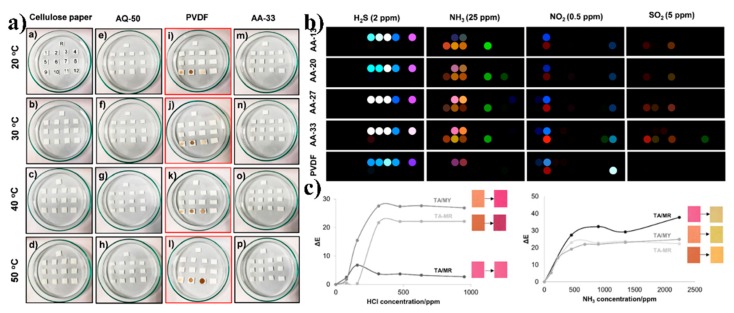
Electrospun NP interfaces for colorimetric sensor: (**a**) Digital photographs acquired in 12 h after chemical exposure at 20–50 °C for bare cellulose paper, AQ-50 substrate(The substrate prepared by this protocol was denoted as an AQ-50 substrate.), polyvinylidene difluoride (PVDF) membrane and AA-33 substrate; (**b**) color difference maps for AA-13 to AA-33 and PVDF-based colorimetric sensor array (CSAs), which include H_2_S, NH_3_, and NO_2_ below their polyelectrolyte (PEL) concentrations and SO_2_ at PEL concentrations [119], copyright 2018 Langmuir; (**c**) color difference between original sample and sample exposed to hydrogen chloride (HCl) (left) and NH_3_ (right) [120], copyright 2016 Wiley-VCH.

Patil et al. demonstrated the fabrication of reusable colorimetric sensing arrays by coating perfluoroalkoxy (PFA) polymer NPs on cellulose filter paper [119]. The key to preparing reusable colorimetric sensor arrays is to prevent particle interactions and probe particle and other chemical particle reactions. On the other hand, the preparation of highly sensitive sensors needs to achieve the maximum possible uniform dispersion of probes. The team pre-diluted the PFA polymer with an organic solvent to reduce particle aggregation, thereby improving the adhesion between the polymer and the cellulose membrane and enhancing the stability of the substrate and probe particles. To verify the chemical stability of the samples, the team compared the product performance under different environments, as shown in Figure 8a. The results showed that substrate, which the initial PFA concentration (w/v%) is 50, designed by the team showed excellent chemical stability. The test results of toxic industrial chemicals showed that all the five samples showed high sensitivity, as shown in Figure 8b. In another case, Geltmeyer et al. developed a colorimetric nanofiber sensor that can be used for the detection of hydrogen chloride and ammonia [120]. Comparing the sensitivity of the nanofiber sensor with different HCl and NH_3_ gas vapor shown in Figure 8c, it is confirmed that when the HCl and NH_3_ gases are excessive, the sensor will produce a more obvious color change that can be visually observed by the naked eye.

### 3.5. SERS Sensing

The regular movement of electrons inside MNPs under the action of external electromagnetic field at a certain frequency produces surface plasma resonance, which greatly enhances the electromagnetic field around the particles and produces some surface enhancement effects, such as SERS. Due to the advantages of high sensitivity, simple sample preparation method, low sample destruction, and consumption, SERS sensing has been widely used in the fields of molecular trace detection, sensing, photocatalytic mechanism, biomedical imaging, and biological detection [121,122].

An important prerequisite for the SERS is a substrate with Raman-enhanced activity. Therefore, the preparation of new-enhanced substrates has become a hot research topic in this field. Many methods have been tried to assemble Ag or Au NPs into special structures to serve as surface-reinforced Raman substrates. The existing methods for preparing substrates include chemical treatment, plate circuit printing, high vacuum deposition, and ordered assembly of NPs. Although the preparation works well, they are both monodisperse substrates. Therefore, the effect is only provided by the outermost layer of the substrate nanostructure, while the longitudinal depth detected by the Raman spectrometer is micron, so the utilization rate of the existing substrate for Raman spectrometer is low. Although the liquid substrate prepared by the sol method can make full use of the detection depth of Raman spectrometer, the effect is affected by the low concentration of NPs in the sol. 

**Figure 9:**
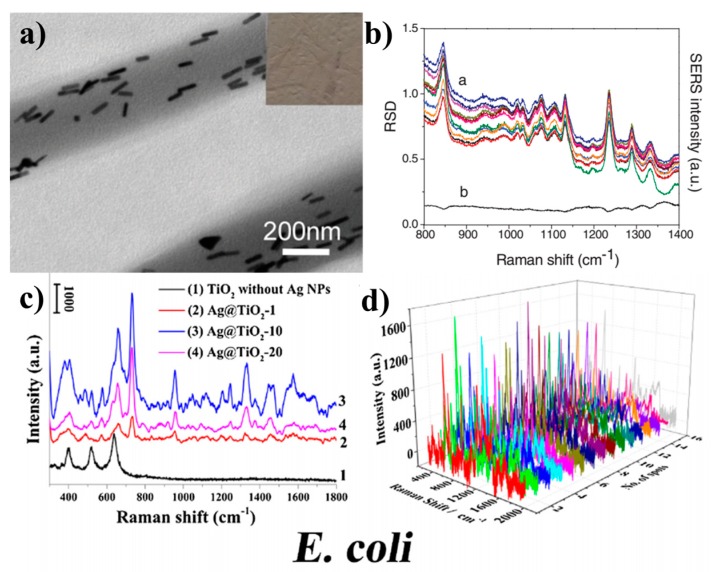
Electrospun NP interfaces for surface-enhanced Raman scattering (SERS) sensing: (**a**) TEM image of AuNRs assembled within the PVA nanofibers; (**b**) relative standard deviation (RSD)-SERS graph (1) SERS spectra of 10^–4^ M 3,3′-diethylthiatricarbocyanine iodide (DTTCI) collected from 12 randomly selected places on the optimized substrate and (2) the corresponding RSD value curve [123], copyright 2012 Wiley-VCH; (**c**) SERS spectra taken from bacteria/Ag@TiO_2_ nanofelts sensing platform with various Tollen’s reagent deposition time (0, 10, and 20 min) and TiO_2_ nanofibers without AgNPs decorated; (**d**) overlapping SERS spectra of bacteria/Ag@TiO_2_-10 nanofibers mats recorded at 16 randomly selected spots for E.coli [124], copyright 2018 Elsevier Ltd.

Electrospinning nanofibers can not only provide dispersed and fixed places for NPs, but also form multilayer dispersed and active nanofiber films. The substrate prepared by this method can effectively increase the contact area between the substrate and the detected molecules, so as to improve the detection sensitivity. For example, Zhang et al. reported an AuNRS/PVA nanofiber mat in which AuNRS were arranged axial along the nanofilaments [123]. Compared with AuNRs, the electrospun mat doped with AuNRs presented a wide absorption band due to the coupling of adjacent nanorods. The nanofiber mats could be used as SERS substrates. Within a certain range, the mat thickness and the content of AuNR increased with the increase of electrostatic spinning time, which resulted in the corresponding increase of Raman peak strength. As shown in Figure 9a,b, 12 sites were randomly selected on the nanofiber felt to obtain the relative standard deviation (RSD) of SERS peak signal strength, which was the parameter to evaluate the reproducibility of Raman spectrum signal, with a value of about 0.1.

In another case, Yang et al. developed an Ag/TiO_2_ electrospinning nanofibrous mat as SERS substrate for direct and sensitive bacterial detection [124]. When it was deposited in Tollen’s reagent for 10 min, it was found that AgNPs were uniformly deposited on TIO_2_ nanofibers. As shown in Figure 9c,d, this kind of nanofiber felt not only has a good SERS performance, but also can be used as SERS substrates for direct detection of E. coli without the need to bind with a bacteria-adaptor. In addition, this kind of fiber felt has excellent antibacterial properties, and has a broad application prospect in the field of biosensors and sterilizations.

### 3.6. Others (Photoelectric, Chemical Resistance)

In addition to the above-mentioned application of electrospinning sensors, in recent years, photoelectric and chemical resistance sensing have also been fabricated by electrospinning. Semiconductor nano devices have unique photoelectric chemical properties, and have been proved to be the preferred materials for transistors, sensors, solar cells, and other applications, especially titanium dioxide with high sensitivity, rapid response and recovery performance. However, the integrated fabrication of semiconductor nanocrystalline electronic devices is still a difficult problem for scientists all over the world. 

**Figure 10:**
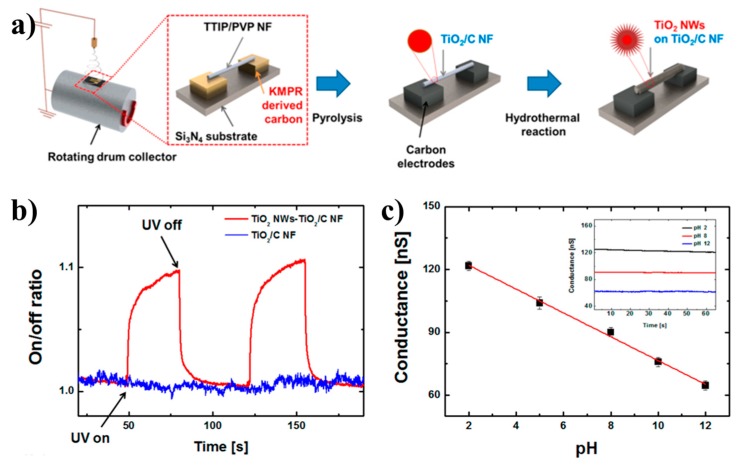
Electrospun NP interfaces for photoelectronic semiconductor sensor: (**a**) Fabrication of hierarchically structured TiO_2_ nanowires (TiO_2_ NWs) on TiO_2_-carbon (TiO_2_/C) NFs suspended and aligned on carbon electrodes; (**b**) and (**c**) applications of hierarchically structured TiO_2_ NWs-TiO_2_/C NF suspended on a carbon-electrode-based electronic device as a ultraviolet (UV) and pH sensor [125], copyright 2014 American Chemical Society.

Electrospinning can deposit nanomaterials directly onto the electrode as shown in Figure 10a and it is noteworthy that this method is relatively simple and low cost [125]. Thus, electrospinning provides a way to integrate nanofibers directly into electrodes without the need for traditional complex processes [126]. For instance, Lee et al. prepared TiO_2_/C nanofibers semiconductor nanodevice by electrospinning, which was further utilized as UV and pH sensors [125]. Moreover, due to the large surface area of TiO_2_ nanowires, the suspended electrode realizes point-to-point contact with the carbon electrode, and this kind of sensor shows very high sensitivity and relatively stable ohmic contact, as shown in Figure 10b. The amorphous structure and more oxygen vacancy defects make TiO_2_/C nanofibers-based devices lose photosensitivity, causing the linear response of semiconductor nanosensors to pH changes, which further confirms the practical application of the fabricated device as a pH sensor, Figure 10c. The development of semiconductor nanosensors provides a new route for the development of other functional nanosensors [127].

Due to its large specific surface area, simple surface modification and good biocompatibility, electrospinning nanofibers have attracted continuous attention in biological sensing detection, drug delivery, and other biomedical fields. However, in the process of biosensor, due to the huge difference in surface energy and energy between NPs and spinning solution, it is easy to cause the agglomeration of NPs and uneven dispersion of particles in the matrix, which greatly limits the sensitivity of the sensor. Zhang et al. appropriately modified the NPs and completed the blending process before electrospinning, during which the matrix material was polymerized, realizing the problem of uniform dispersion of NPs in the polymer matrix and ultimately improving the detection performance of the biosensor [128]. Due to its inherent characteristics, the electrostatic spinning technology will further display its advantages in biological sensing and semiconductor nano-device design. The applications mentioned above are summarized in Table 2.

## 4. Conclusions and Outlooks

In summary, we demonstrated and discussed the electrospinning fabrication and sensor applications of various NPs-based materials interfaces. Based on the above case studies, it can be found that the in-corporation of metal, metal oxide, alloy, and carbon NPs into electrospun fibrous polymer matrix caused in the creation of various functional materials interfaces, in which the polymer matrix (such as conductive polymers), nanoporous structure of materials, and bound NPs exhibited synergistic effects towards the final performance of the fabricated sensor devices. Therefore, the fabricated NPs-based materials interfaces have shown promising applications as sensors and biosensors by combining the fabricated materials interfaces with electrochemistry, electronics, fluorescence, colorimetric, SERS, and other detection techniques together. Compared to other methods for creating functional materials for sensors, electrospinning reveals several advantages such as quicker production, lower cost, scale-larger synthesis, more flexibility, and wider applications.

Although great achievements on the fabrication and sensor applications of electrospun materials interfaces have been obtained, in our opinion there are still some spaces that could be filled in. First, hybrid fibers with high orientation can be obtained by changing the receiving device, controlling electric field, and adding magnetic field. In addition, the fibrous membranes with the 2D spider network structure can be constructed by regulating the electric field, solution property, and environmental parameters. Second, more focus could be paid onto the fabrication of conductive polymers and NPs-based materials interfaces for fabricating label-free sensors and biosensors. Third, micro/nano fibers with special structures such as ribbon, spiral, porous, necklace, multi-core, shell, and hollow can be prepared for fabricating sensors. Fourth, NPs-based materials interfaces can be further modified with other biomolecules such as enzyme, antibody, antigen, DNA, protein, aptamer, and others to improve their applications as biosensors. Fifth, it is possible to create some new functional materials by using graphene, graphene quantum dots, MoS_2_, and other 2D materials through electrospinning, which will promote the design and fabrication of novel sensors with improved performances.

## Figures and Tables

**Table 1:** Summary on the electrospinning methods for fabricating various NP-based materials interfaces.

Interfaces	Method	Material	Advantages	Disadvantages
MNP-based	blending method	Ag/Au/Pt@ polymer	Briefness, high yield	Agglomeration, Surface barren
Oxide NP	post-treatment	ZnO, In_2_O_3_, NiO, Al_2_O_3_	High-activity	Complexity, instability
Alloy/metal oxide NP	blending method, post-treatment	Pd^0^-SnO_2_, NiO@ZnO, Pt-Pb	Ditto	Ditto
Carbon NP	post-modification		High-activity, Homogeneous distribution	Homplexity

**Table 2:** Summary on the electrospun materials interfaces for various sensors.

Type of Sensor	Detection Object	Electrospun NP-Based Interfaces	Detection Limit	Linear RANGE	Ref.
	Glucose	Ni/CoO-CNF	0.03 μM	0.25–600 μM	[95]
Electrochem	AZA	Ag/PVA/CS	0.09 μM	0.14–2.88 μM	[96]
sensors	H_2_O_2_	PtNi/CeO_2_/NCNF	0.025 μM	0.5 μM–15 nM	[63]
	Pb^2+^	Ag NPs-PA 6-CNW	10 nmol L^−1^	-	[101]
Electronic sensors	N-butanol	Au-WO_3_ NF	-	-	[108]
	(C_2_H_5_)_3_N	PAN-NPs	-	-	[111]
Fluorescent sensors	H_2_O_2_	PVA/GQD NF	0.61 μM	0.1 mM–75 mM	[115]
	Hg^2+^	poly(HEMA-co-NMAco-RhBN2AM)	10^−3^ M	10^−3^–10^−7^ M-	[117]
Colorimetric sensors	H_2_S, NH_3_, NO_2_	PFA- cellulose	2, 25, 0.5 ppm	-	[119]
	HCl, NH_3_	TA/MY	220 ppm	-	[120]
pH	pH	TiO_2_/CNF	5.68 ± 0.28 nS/pH -	-	[125]
